# Research progress on vestibular dysfunction and visual–spatial cognition in patients with Alzheimer’s disease

**DOI:** 10.3389/fnagi.2023.1153918

**Published:** 2023-04-20

**Authors:** Yan Huang, Jiaxi Xu, Xuehao Zhang, Yuhe Liu, Enyan Yu

**Affiliations:** ^1^School of Medical Technology and Information Engineering, Zhejiang Chinese Medical University, Hangzhou, China; ^2^The Second School of Clinical Medicine, Zhejiang Chinese Medical University, Hangzhou, China; ^3^Department of Otolaryngology, Head and Neck Surgery, Beijing Friendship Hospital, Capital Medical University, Beijing, China; ^4^Cancer Hospital of the University of Chinese Academy of Sciences, Zhejiang Cancer Hospital, Hangzhou, China

**Keywords:** Alzheimer’s disease, cognition, rehabilitation, vestibular dysfunction, visual–spatial cognition

## Abstract

Alzheimer’s disease (AD) or vestibular dysfunction may impair visual–spatial cognitive function. Recent studies have shown that vestibular dysfunction is increasingly common in patients with AD, and patients with AD with vestibular impairment show more visual–spatial cognitive impairment. By exploring the relationship and interaction mechanism among the vestibular system, visual–spatial cognitive ability, and AD, this study aims to provide new insights for the screening, diagnosis, and rehabilitation intervention of patients with AD. In contrast, routine vestibular function tests are particularly important for understanding the vestibular function of patients with AD. The efficacy of vestibular function test as a tool for the early screening of patients with AD must also be further studied. Through the visual–spatial cognitive ability test, the “spatial impairment” subtype of patients with AD, which may be significant in caring for patients with AD to prevent loss and falls, can also be determined. Additionally, the visual–spatial cognitive ability test has great benefits in preventing and alleviating cognitive decline of patients with AD.

## Introduction

Alzheimer’s disease (AD) is a progressive neurodegenerative disease with a hidden onset. In 2018, the Alzheimer’s Disease International estimated that the global prevalence of dementia was approximately 50 million, which is expected to triple by 2050. Two-thirds of these individuals live in low-and middle-income countries (Scheltens et al., 2021), which has a huge impact on public health (2021 Alzheimer’s Disease Facts and Figures, 2021). Cognitive disorders, including memory disorders, impairment of visual–spatial skills, and executive dysfunction, often present as a clinical manifestation of AD. Moreover, the etiology of AD remains unclear (van der Flier et al., 2011).

The vestibular system plays an important role in body balance and maintaining the stability of movement. In addition to providing input information for brainstem reflexes, the vestibular system also sends projection signals to the subcortical and cortical structures, in which information about the head direction and movement is used in advanced cognitive processes, such as spatial memory and navigation (Bigelow and Agrawal, 2015; Smith, 2017). Loss or disorder of the vestibular sensory function is one of the reasons for visual–spatial cognitive impairment. Increasing evidence shows that a decline in vestibular function is related to poor spatial cognitive ability in healthy older adults (Bigelow et al., 2015). Zhang et al. (2022) found that (i) visuospatial ability may decline with age, (ii) older adults have weaker visuospatial cognition than younger adults, and (iii) older patients with vestibular dysfunction have worse visuospatial cognition than normal older adults.

Patients with AD differ, on average, from each other on a number of clinical, neuropsychological, neuroimaging, and neuropathological variables. Some patients with AD tend to have worse ideomotor praxis and visual–spatial skills (Palasí et al., 2015; Joubert et al., 2016). The degree of vestibular damage in patients with AD is higher than that in healthy older adults of the same age (Previc, 2013). Therefore, there is a certain relationship between vestibular dysfunction and AD. This article discusses the relationship and interaction mechanism between the vestibular system, visual–spatial cognitive ability, and AD to provide information for the screening, diagnosis, and rehabilitation intervention in patients with AD.

### Vestibular dysfunction in patients with AD

Vestibular dysfunction is becoming increasingly common in patients with AD, with a study showing an 85.19% probability of cervical vestibular evoked myogenic potential (cVEMP) failing to elicit a distinct waveform bilaterally at a tone burst of 500 Hz and 125 dB SPL in patients with AD (Wei et al., 2018). Wei et al. (2019) evaluated the vestibular physiological function of 51 patients with AD and 295 normal controls using cVEMP, ocular vestibular evoked myogenic potentials, and video head impulse test (v-HIT). It was found that, compared with the normal control group, the probability of vestibular function impairment increased by at least three to four times in patients with AD. Thus, there is increased attention to whether vestibular function tests can be used as a means of early dementia screening. Bosmans et al. (2021) reviewed seven articles and analyzed the vestibular function test results of 150 patients with AD and 481 older adults with normal cognition as controls. The vestibular tests included the v-HIT, caloric test, and cVEMP. The results showed that the latency of P13 in patients with AD was significantly longer than that in the normal control group, and the amplitude of cVEMP was significantly lower than that in the normal control group. This study suggests that cVEMP may be used as a screening tool to distinguish patients with AD from older adults with normal cognitive function. However, more clinical trials are needed to verify the sensitivity of v-HIT in screening patients with AD.

The mechanism underlying the higher incidence of vestibular dysfunction in AD patients is unclear. The degeneration of the cholinergic system in the posterior parietotemporal region, medial temporal region, and posterior cingulate region can occur at an early stage of AD disease progression. The progression of AD disease also affects the function of the hippocampus, temporoparietal junction, insular cortex, and dorsal thalamus, which are related to the input of vestibular information (Previc, 2013; Agrawal et al., 2020). Amyloid-β (Aβ) protein, a macromolecular substance derived from the fat membrane of nerve cells, is the main pathological deposit in patients with AD. Accumulation of Aβ in the central vestibular pathway may damage the physiological function of the vestibule (Kamil et al., 2018). Other risk factors of AD, such as age, cardiovascular disease, diabetes, and traumatic brain injury, may also be related to vestibular dysfunction (Oron et al., 2017; Chen et al., 2021).

### Visual–spatial cognitive impairment in patients with AD

In addition to prominent memory problems, Alzheimer’s disease is characterized by visual–spatial perception dysfunction (Mendez et al., 1990; Rizzo et al., 2000). Visual–spatial cognitive ability includes spatial memory, spatial navigation, and mental rotation. Spatial memory refers to the process by which the human body uses visual information or other sensory information, such as vestibular, auditory, and proprioceptive, to sort out, encode, process, and store surrounding environmental information. Successful spatial navigation depends on good spatial memory in the early stage (Iachini et al., 2021). Spatial navigation is the ability of an individual to identify the current location and environment and navigate to the next destination. Spatial navigation is a basic behavioral ability of human beings, which integrates various cognitive abilities, such as memory, execution, and perception, and helps them choose and apply the corresponding navigation strategies. Navigation strategies include two types: allocentric strategy and egocentric strategy. The allocentric strategy relies on landmarks and signs for spatial positioning and navigation; the egocentric strategy mainly depends on the main body orientation and geographical spatial clues (such as the orientation of the sun or moon) (Stewart et al., 2022). In the real-world, these two strategies can be used separately or jointly. Mental rotation refers to the rotation and direction change of different dimensions in the brain according to the representation of objects or graphics (Stewart et al., 2022). Currently, the evaluation scales and means used to evaluate visual–spatial cognitive ability in clinics mainly include the card rotations test, Benton visual retention test, money road map test (MRMT), Corsi block tapping task, and virtual Morris water task (MWT).

AD is characterized by the presence of extracellular amyloid plaques and intracellular neurofibrillary tangles. Neuroinflammation and synaptic and neurotransmitter loss also participate in the pathogenesis of AD. Clinically, patients’ memory loss and related cognitive dysfunction are the main characteristics of AD. According to the age of onset, AD can be further divided into two subtypes: early onset and late onset (Mendez, 2017; Di Resta and Ferrari, 2019; Ayodele et al., 2021). High permeability mutations in β-amyloid precursor protein, recombinant presenilin 1, and recombinant presenilin 2 lead to autosomal dominant early onset (Sun et al., 2017). Many patients with early-onset AD have a gradual decline in visuospatial skills, known as posterior cortical atrophy or “Benson’s disease.” In 1988, Benson et al. (1988) described 83 patients with this syndrome with complex visual symptoms, including dyslexia, perceptual visual agnosia, Bahrain syndrome (simultaneous agnosia, visual ataxia, optokinetic eye muscle apraxia), visual–spatial positioning difficulties, Gerstmann syndrome, and possible left visual field defect and visual structure test disproportional damage (Mendez, 2017). Several patients with AD have an obvious decline in visuospatial skills.

The plaques and tangles of abnormal AD proteins spread to the different regions of the brain, destroy brain function, and cause multi-dimensional cognitive impairment, including visual–spatial cognition. Reliable prediction indicators of amyloidosis *in vivo* have been reported in the literature (Dubois et al., 2014). However, they are often limited to the research environment due to cost, availability, or patient safety. Before the development of amyloid biomarkers, the clinical diagnostic criteria for diagnosing AD mainly depended on neuropsychological tests (Blessed et al., 1968; Chapman et al., 2011; Burrell and Hodges, 2015). In routine clinical practice, some cognitive function test scales, such as the Mini-Mental State Examination (MMSE) and Montreal Cognitive Assessment, are used to assess the overall cognitive ability of patients with AD.

However, in some current studies, the accuracy of the visuospatial cognitive correlation test in screening patients with AD may be better than that of conventional global cognitive tests. Plácido et al. (2021) recruited 72 older people aged 60 years and above (39 healthy people and 43 with AD) to assess their spatial navigation ability using the floor maze test (FMT) and assessed their overall cognitive ability using the MMSE. The results showed that, compared with the MMSE test, FMT had better sensitivity in distinguishing patients with AD from healthy peers, indicating that the decline in visual–spatial cognitive ability could be independent of the decline in general cognitive ability. In addition, visual–spatial cognitive impairment may be an important cognitive marker for recognizing patients with early AD (Lester et al., 2017). Parizkova et al. (2018) studied the preferences of patients with AD regarding navigation strategies. With an increase in AD severity, the patient’s preference for self-centered strategies was higher than that for object-centered navigation strategies. Atrophy of the hippocampus and basal forebrain in patients with AD may lead to the reduction of object-centered navigation strategies, some of which are replaced by self-centered navigation strategies. This obstacle can be detected using a simple visual–spatial cognition questionnaire (Cerman et al., 2018). Salimi et al. (2019) used Addenbrooke’s Cognitive Examination-Revised (before 2014) and Addenbrooke’s Cognitive Examination-III (after 2014) for cognitive screening, and found that the visual–spatial ability of patients with AD was impaired in all tests. The visual space defect of patients with AD is manifested as the impaired performance in the Addenbrooke’s Cognitive Examination visual space subscale, Rey-Osterrieth complex figure copy, and visual object and space perception battery point count and position discrimination subtasks. It supports the view that visuospatial dysfunction is a prominent early feature of clinically possible AD.

Vogel et al. (2021) used positron emission tomography imaging technology to monitor the accumulation of tau protein in the brains of 1,612 patients. They delineated four different subtypes of AD pathology: (1) subtype 1, wherein tau protein is mainly deposited in the temporal cortex, affecting memory, accounting for 33%; (2) subtype 2, wherein tau protein is mainly deposited in the occipital cortex, affecting visual–spatial processing, accounting for 30%; (3) subtype 3, wherein tau protein spreads asymmetrically in the left brain, affecting language ability, accounting for 19%; and (4) subtype 4, wherein tau protein deposits and spreads in other parts of the cerebral cortex, affecting executive function, accounting for 18%. This explains why different patients with AD may have different symptoms. Wei et al. (2018) recruited 28 patients with AD and measured their cVEMP. The MRMT and trail-making test B were used to assess visual–spatial cognitive ability. According to the MRMT test results, patients with AD were divided into “space normal” and “space impaired” groups. The results showed that the rate of cVEMP unreduced in the “space impaired” AD group was significantly higher than that in the “space normal” AD group. This also indirectly indicates that there may be a specific “space impaired” subtype in patients with AD.

### Relationship among AD, vestibular system, and visual–spatial cognition

Visual–spatial cognition involves the fusion and coding of multiple sensory organs, of which visual nerve conduction and coding are the main ones. Nerve conduction mainly includes the dorsal and ventral pathways, which cooperate to complete the perception and control of the spatial environment (Zou, 2014). The dorsal pathway originates from the primary visual cortex V1 of the occipital lobe and reaches the parietal lobe and other related brain regions through MT, which is responsible for processing the visual and perceptual information related to movement. The ventral pathway also comes from the primary visual cortex V1 of the occipital lobe and reaches the lower temporal lobe and other related brain regions through V4, which is responsible for processing color, shape, and other related information (Bao et al., 2017). Visual–spatial information is transmitted layer-by-layer through the visual transmission pathway, and the information extracted from the changing visual images by the relevant functional brain regions also changes from simple to abstract and complex. In addition to the advanced visual conduction pathway, the vestibular thalamic cortical pathway affects the processing of spatial cognition and spatial recognition and navigation through vestibular information (Hitier et al., 2014). When these neural conduction networks and the cortex related to spatial processing are abnormal, the visual–spatial cognitive ability will have corresponding obstacles.

Vestibular information provides basic clues regarding spatial orientation, such as eye movement control, posture control, balance, and orientation. In addition to traditional low-level reflex motor circuits, the vestibular system is associated with higher levels of cognitive function. There are four main reflex pathways from the vestibule to the cortex, including the vestibulo-cerebello-cortical pathway, vestibulo-thalamo-cortical pathway, head direction pathway, and theta pathway (Hitier et al., 2014). Vestibular information can be transmitted to the parietal lobe through the thalamus and further to the entorhinal cortex or hippocampus to complete the recognition of environmental spatial information. Loss of vestibular information input may damage these cognitive and emotional circuits. Brandt et al. (2005) performed magnetic resonance imaging on 10 patients with a chronic bilateral vestibular disorder, and the results showed that the hippocampus of patients with chronic bilateral vestibular disorder showed significant atrophy compared with the normal control group. Hippocampal atrophy may reflect a neuroanatomical correlation between poor spatial cognitive ability and vestibular dysfunction.

The amyloid cascade hypothesis and tau protein pathogenesis theory are the two main pathogeneses of AD (Sun et al., 2017; Khan et al., 2020). The amyloid cascade hypothesis refers to Aβ, wherein the abnormal metabolism of tau leads to an increase in its production, which leads to changes in tau protein hyperphosphorylation, neuronal damage, and oxidative stress, ultimately leading to the impairment of cognitive function. The pathogenesis of the tau protein is related to intracellular neurofibrillary tangles that form as tau accumulates, which directly damages neurons. The first area affected by AD is the hippocampus, which is primarily responsible for general memory and spatial storage. With the development of the disease, abnormal proteins are deposited and tangled in the occipital visual cortex and related high-level visual–spatial processing pathways, which further aggravates spatial cognitive impairment.

Both the visual–spatial cognitive impairment caused by vestibular dysfunction and visual–spatial cognitive impairment caused by AD seem correlated with the hippocampus. Atrophy of the hippocampus may affect the ventral cortical conduction pathway of visual processing and the corresponding vestibular thalamic cortical pathway. With the aggravation of AD, abnormal proteins may further deposit and tangle in the different cortical and brain regions, causing ventral and dorsal visual–spatial conduction pathways, as well as more abnormalities in the cortical regions related to spatial processing. Therefore, compared with the visual–spatial cognitive impairment caused by vestibular dysfunction, the mechanism of visual–spatial cognitive impairment caused by AD may be more complex. Some patients with AD have a worse visuospatial cognitive ability due to vestibular dysfunction. Wei et al. (2017) recruited 39 patients with AD and measured their cVEMP to evaluate balloon function. A visual–spatial questionnaire survey was conducted among all patients to assess whether they had obstacles in space driving and other aspects. The results showed that, compared with patients with AD with normal balloon function, patients with AD with bilateral balloon damage were 12 times more likely to have difficulty driving.

### Improvement of cognitive ability of patients with AD through vestibular rehabilitation

Falls are the main medical and health problem faced by patients with AD and their caregivers. Vestibular dysfunction is a known risk factor for falls. The prevalence of vestibular dysfunction in patients with AD is higher than that in the age-matched control group (Wei et al., 2017). Thus, vestibular physiotherapy (VPT) improves the balance of patients with vestibular dysfunction and normal cognitive function and reduces the risk of falls. Guidetti et al. (2020) evaluated 263 patients with chronic bilateral and unilateral vestibular dysfunction and 430 healthy individuals using the Corsi building block test. The results showed that the correct rate of the Cauchy building block test in patients with vestibular dysfunction was significantly lower than that in healthy controls. However, 5 days after the vestibular rehabilitation training, the same group of patients was tested for the Cauchy bricks test. The repetition accuracy rate of patients was improved compared to that before rehabilitation training, and their memory ability was improved to some extent. However, the effectiveness of VPT in improving the balance and falls of patients with AD with vestibular dysfunction requires further study. Some scholars have suggested that the theoretical framework of VPT rehabilitation and motor learning should be appropriately improved in patients with cognitive impairment (Klatt et al., 2019).

Vestibular nerve stimulation can promote the prevention and recovery of many diseases, such as dementia, stress-related mental disorders, and neurodegenerative diseases (Jagadeesan et al., 2021). According to different stimulation types, vestibular nerve stimulation can be divided into types such as vestibular electrical stimulation (GVS), vestibular thermal stimulation, and vestibular rotation stimulation. GVS can transmit vestibular information to the hippocampus through the basal ganglia pathway and participate in the regulation of spatial learning and memory. Nguyen et al. (2021) conducted GVS treatment in mice with unilateral labyrinthectomy and found that it improved their spatial memory and navigation ability. Adel Ghahraman et al. (2016) injected streptozotocin into the brains of rats to build a cognitive impairment model. The rats were divided into three groups and received GVS with low amplitude noise one or five times, each time lasting for 30 min, or did not receive GVS at all. MWT was conducted in each group to evaluate spatial memory and spatial navigation ability. The results showed that rats that received GVS treatment five times had better performance in MWT tasks, and memory impairment was significantly improved. This effect may be partly attributed to the frequent activation of vestibular neurons and their associated hippocampi by GVS.

AD is a long-term disease that may take several years or decades from neuropathological injury to complete loss of self-care ability. At different stages of disease progression, corresponding rehabilitation and nursing measures must be formulated and implemented according to the current concerns, and comprehensive prevention and treatment must be performed (Yang and Jia, 2021). The comprehensive and systematic cognitive assessment of patients is conducive to the formulation of appropriate rehabilitation intervention programs; however, the assessment of cognitive impairment for clinical diagnosis mainly focuses on the cognitive impairment mode of AD, while the rehabilitation assessment of AD requires a comprehensive assessment of various functional disorders that may exist in patients, such as daily behavior activity ability, hearing, and vestibular function, which are not limited to cognitive function (Chinese Expert Consensus on Rehabilitation Management of Alzheimer’s Disease 2019, 2020).

### Outlook

AD and other forms of dementia are major and increasingly serious global health challenges. Currently, 40–50 million people worldwide suffer from dementia (Prince et al., 2013; Wu et al., 2017), which places a heavy burden on society (GBD, 2019). Care and support for patients with dementia has a wide impact on families, medical care systems, and society (Etters et al., 2008). There are currently no strategies to cure AD or change its course. However, early screening and diagnosis are conducive to appropriate treatment measures for patients with AD. In addition to aging, many risk factors, such as microvascular disease and hearing loss, are associated with cognitive decline (Livingston et al., 2020). With the incidence rate of cognitive impairment-related diseases, such as AD, increasing gradually, the intervention and control of various risk factors become particularly important. Vestibular injury is particularly common in patients with AD, especially in those with visual–spatial cognitive impairment. Therefore, routine vestibular function tests are particularly important for understanding the vestibular function of patients with AD. The efficacy of the vestibular function test as a tool for the early screening of patients with AD must also be further studied. Through the visual–spatial cognitive ability test, the “spatial impairment” subtype of patients with AD, which may be significant in caring for patients with AD to prevent loss and falls, can also be determined and has great benefits in preventing and alleviating the cognitive decline of patients with AD.

## Author contributions

YH and XZ: ideas, preparation, specifically writing the initial draft. JX: formulation or evolution of overarching research goals and aims. YL and EY: ensure that the descriptions are accurate and agreed upon by all authors. All authors contributed to the article and approved the submitted version.

## Funding

This study was supported by the National Natural Science Foundation of China (grant no. 8177051246).

## Conflict of interest

The authors declare that the research was conducted in the absence of any commercial or financial relationships that could be construed as a potential conflict of interest.

## Publisher’s note

All claims expressed in this article are solely those of the authors and do not necessarily represent those of their affiliated organizations, or those of the publisher, the editors and the reviewers. Any product that may be evaluated in this article, or claim that may be made by its manufacturer, is not guaranteed or endorsed by the publisher.
